# Molecular detection of *Paracoccidioides* in soil from an urban area of southern Brazil

**DOI:** 10.1590/0037-8682-0172-2019

**Published:** 2020-03-16

**Authors:** Josiara Furtado Mendes, Vanice Rodrigues Poester, Andrea Von Groll, Mário Carlos Araújo Meireles, Melissa Orzechowski Xavier

**Affiliations:** 1 Universidade Federal de Pelotas, Programa de Pós-Graduação em Veterinária, Pelotas, RS, Brasil.; 2 Universidade Federal do Rio Grande, Programa de Pós-Graduação em Ciências da Saúde, Rio Grande, RS, Brasil.; 3 Universidade Federal do Rio Grande, Faculdade de Medicina, Laboratório de Micologia, Rio Grande, RS, Brasil.

**Keywords:** Paracoccidioidomycosis, Paracoccidioides brasiliensis, Environment

## Abstract

**INTRODUCTION:**

Previous studies that detected *Paracoccidioides* spp. DNA in soil taken from rural areas have shown this to be a valuable tool for ecological and epidemiological studies. This study reports the detection of *Paracoccidioides* spp. DNA in soil samples from an urban area of southern Brazil.

**METHODS:**

Sixteen soil samples were submitted to nested-PCR and the amplicons of a representative number of positive samples were sequenced.

**RESULTS:**

*Paracoccidioides* spp. DNA was found in 44% of samples. Four DNA amplicons were sequenced, showing 100% homology with *P. brasiliensis*.

**CONCLUSIONS:**

The southern Brazilian urban population is commonly exposed to the *Paracoccidioides* fungus.

Paracoccidioidomycosis (PCM) is a granulomatous systemic disease that is caused by the thermodymorphic fungi of the genus *Paracoccidioides*. This mycosis is the second most common endemic mycosis in Latin America, and has a significant impact on public health in endemic areas^1^. Brazil accounts for approximately 80% of all PCM cases and the disease is responsible for the hospitalization of 7.99/1000 hospital patients in the country^1^. 

PCM has expanded geographically, and is no longer confined to rural workers. It is described as being quite prevalent in urban patients who do not have any involvement in agricultural activities, including in HIV patients^2^. Epidemiological surveys support the hypothesis that the fungus is no longer restricted to rural zones due to factors such as increased urbanization, demographic growth, and deforestation^2^ ^,^ ^3^.

*Paracoccidioides* spp. are present in soil; however, due to their slow growth they have infrequently been isolated from this environment. Several literature reports express concern that isolation by culture methods have a high failure rate, necessitating alternative methods to determine the epidemiology and infection source of PCM^3^ ^,^ ^4^. One of these uses molecular methods to detect the fungus in soil^3^. In Brazil, the states of Goiás (GO), Minas Gerais (MG), Rondônia (RO), and Rio Grande do Sul (RS) have reported the presence of this fungus in the environment using molecular methods^3^ ^,^ ^5^.

PCM is endemic to RS state, Brazil, and serological studies suggest the presence of *Paracoccidioides* spp*.* in urban and rural populations in the southern region^6^ ^-^ ^10^. More recently, Mendes et al.^5^ reported the detection of *Paracoccidioides* spp. DNA in the Pampa Biome. However, the presence of this fungus is still undetermined in urban areas; consequently; this evaluation is important for fostering a better understanding of the regional PCM epidemiological context. Therefore, this study aimed to evaluate the presence of *Paracoccidioides* spp. DNA in soil samples collected from an urban area in a city from southern RS, Brazil.

Soil samples were collected in Rio Grande City, which is a municipality located on the coast of southern RS, Brazil. Rio Grande City has an area of 2,817 km^2^, and has a subtropical climate, with an average annual temperature of 18.2 °C. The atmospheric relative humidity is approximately 80%, and rainfall is abundant throughout the year, which may be affected by the direct influence of the El Niño and La Niña phenomena^11^.

Sixteen samples comprising 50-100 g of soil were collected from an area of approximately 2 m², as described by Moura et al.^12^ from 12 different districts of the city. Samples were obtained by scraping the soil with sterile spatulas. Each soil sample was stored in an individual sealed container, which was stored in a bigger container that contained only sealed soil samples to avoid environmental contamination, at room temperature (20 - 27 °C) and humidity (82 - 88%) for a period of six months to one year before DNA extraction. Fungal genomic DNA was extracted from these soil samples using a Soil DNA Isolation Kit (Norgen Biotek Corp^®^, Thorold, Canada), following the manufacturer's instructions. DNA samples were quantified by spectrophotometer (NanoVue Plus^TM^, Biochrom, Holliston, MA, USA) and confirmed by electrophoresis on a 0.8% agarose gel. 

The molecular detection of *Paracoccidioides* spp. DNA was performed using a nested-PCR using the panfungal primers ITS4 and ITS5 for the external region, and PBITS-E and PBITS-T for the internal amplified region^13^. In order to avoid contamination, the DNA extraction was performed in a flow cabinet, in a different room, and on different days than the PCR. In addition, the PCR solution was prepared in a separate room from the room where the addition of the DNA and the amplicons were performed. In addition, preventive measures were taken, such as, using an ultra violet lamp, different laboratory coats in different rooms, and tips with a filter, and disinfection of the micropipettes with 70% alcohol. With the aim to minimize the risk of false positives, the nested-PCR technique was replicated at least three times, and for each round of PCR, the sequence of the samples tested was randomized. Amplification was performed in a thermal cycler (Mastercycler, Eppendorf^®^, Hamburgo, Germany). Genomic DNA from a clinical isolates of *P. brasiliensis* (Pb18) and *Sporothrix* sp. were used as positive and negative controls, respectively. The presence of a 424 bp DNA amplicon was verified by electrophoresis on a 1.5% agarose gel, stained with GelRed^TM^ (Biotium, Hayward, CA, USA), and visualized on a UV light transluminator. A 100 bp molecular ladder (Ladder 100pb, Ludwig Biotec^®^, Alvorada, Brazil) was used to determine the size of the DNA amplicon.

In order to confirm the specificity of the DNA amplified, a representative number of positive samples were sequenced (ACTGene Análises Moleculares Ltda, Porto Alegre, RS, Brazil) using the automatic sequencer ABI-Prism 3500 Genetic Analyzer armed with 50 cm capillaries and POP7 polymer (Applied Biosystems^TM^, Foster City, CA, USA), with 2.5 pmol of the primer (PBITS-E) and 0.5 mL of BigDye^TM^ Terminator v3.1 (Thermo Fisher Scientific^TM^, Waltham, Massachusetts, USA) in a final volume of 10 mL. Resulting sequence data collection files were analyzed using MEGA software version 5.2.2, in which low base quality was manual evaluated. The undefined initial bases (approximately 25 nucleotides) were removed from the sequences, and undefined nucleotides were replaced with the representative letter according to the predominant base. Species homology was defined by comparing the nucleotide sequences using BLAST on the NCBI website (https://blast.ncbi.nlm.nih.gov/Blast.cgi).

A map was created with MATLAB software (MathWorks®, Natick, Massachusetts, USA), project the sample geographic position on a Google Map Image.

Of the 16 samples analyzed, an amplicon of approximately 424 pb compatible with *Paracoccidioides* spp. DNA, was visualized in seven samples, corresponding to a positivity rate of 44% (Figure 1 and Figure 2). All of the four amplicons that were sequenced were identified as *P. brasiliensis,* with 100% DNA homology to strain CBS 372.73 (GenBank: MH860706.1). The nucleotide sequences were deposited in the GenBank database (accession numbers: MN271899, MN271900, MN271901, and MN271902).

**FIGURE 1:**
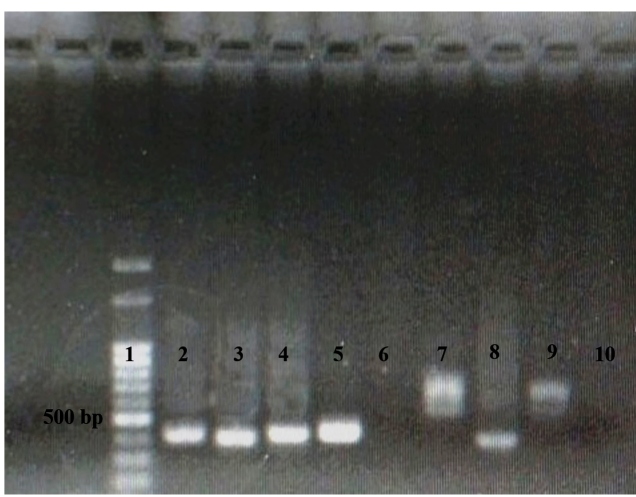
DNA electrophoresis on a 1.5% agarose gel of the nested-PCR amplicon from seven soil samples. **Lane 1:** 100 bp size standard; **lanes 2, 3, 4, and 5:** samples positive for *Paracoccidioides* spp.***,*** showing an amplicon of approximately 424 bp; **lane 6 and 7:** negative samples without a 424 bp amplicon; **lane 8:** positive control (***Paracoccidioides spp.)*** ; **lane 9:** negative control (*Sporothrix* spp. genomic DNA), showing an amplicon of approximately ~800-900 bp, originating from the first round of PCR with panfungal primers; **lane 10:** blank (reagent mix without any DNA).

**FIGURE 2:**
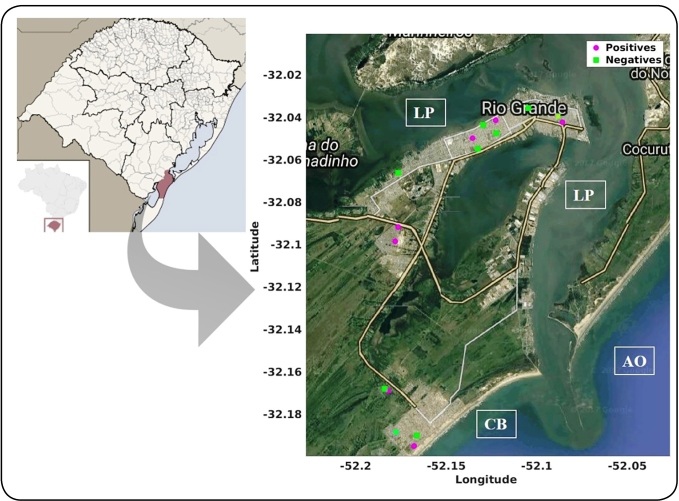
Geographic location of samples that were positive (pink markers; n = 7) and negative (green markers; n = 9) for the presence of *Paracoccidioides* spp. DNA in the City of Rio Grande, southern Rio Grande do Sul, Brazil. **CB:** Cassino beach; **AO:** Atlantic Ocean; **LP:** *Lagoa dos Patos* (Patos Lagoon).

This study represents the first detection of *P. brasiliensis* DNA present in the soil of an urban area of southern Brazil. In addition, we found positive samples in seven different neighborhoods, suggesting that the fungus is widely dispersed throughout the city. Although the DNA of the 16 samples were extracted at the same time, negative controls were used in all of the PCR techniques performed. In addition, it is important to reinforce that preventive measures were taken to avoid contamination. These data corroborate a seroepidemiological investigation conducted by Teles et al.^9^, which demonstrated a high rate of PCM infection in urban dogs from this region. The high positivity rate in the soil samples suggests that the local population is considerably exposed to *Paracoccidioides* spp., and consequently, is at risk of infection. In fact, more than 100 cases of PCM have been reported from this region in a study by Souza et al.^6^, the highest figure in the last two decades.

Rain is well distributed through the year in the region of the study, and often it is influenced by the El Niño phenomenon, with consequently more frequent and abundant rainfall. Although the winters are severe, with low temperatures, the annual average temperature is approximately 18 °C. The sandy soil and the high rates of humidity throughout the year are compatible with the description of the ideal characteristics for the growth and development of *Paracoccidioides* ^4^ ^,^ ^11^ ^,^ ^13^. In addition to this, the geographical peculiarity of this region, characterized as a coastal city surrounded by the major lagoon of South America (Patos Lagoon), an estuarine lagoon (Mirim Lagoon), and 250 km of coastline from the Atlantic Ocean (Cassino Beach)^11^, may have contributed to the high positivity rate in the soil samples.

A search of the PubMed database using the descriptors [*Paracoccidioides* and soil] resulted in 63 articles, in which only five showed the detection of *Paracoccidioides* spp. in soil from Brazil^3^ ^,^ ^4^ ^,^ ^5^ ^,^ ^14^ ^,^ ^15^. Two of these used culture methods and had positivity detection rates between 0% and 0.6%^4^ ^,^ ^14^, two used animal inoculation and had detection rates between 0% and 0.13%^4^ ^,^ ^15^, and three used molecular methods resulting in detection rates between 35% and 83%^3^ ^,^ ^4^ ^,^ ^5^. These reports highlight the higher sensitivity of molecular techniques for *Paracoccidioides* spp. detection. In line with this, Arantes et al.^3^ found a detection rate between 35% and 75% for molecular detection of *P. brasiliensis* and *P. lutzii* in the states of GO, RO, and MG, while in our study, 44% of samples were positive for *P. brasiliensis* in an urban area of RS.

Some limitations of our study include retrospective characteristics which resulted in a prolonged storage time for some samples, the lack of analysis using culture and animal inoculation to compare with the molecular detection rate, and the absence of results concerning *P. brasiliensis* and *P. lutzii* DNA differentiation in the electrophoresis analyzes. Therefore, further studies should be performed in the same region, using whole genome analyses of soil isolates to improve the methods and molecular techniques employed in this study. These data could be integrated into a database concerning the ecological and geographic distribution of *Paracoccidioides* spp. in Brazil.

Souza et al.^6^ demonstrated that the mean period leading to the diagnosis of PCM was greater than one year. This information, associated with the presence of *Paracoccidioides* spp. in rural^5^ and urban areas of southern Brazil as confirmed by our results, suggests that PCM is a neglected disease in this region, and its prevalence is probably underestimated. Further studies with a PCM clinical-epidemiological focus are necessary to confirm this. However, measures aimed at improving diagnostic support and promoting the training of health professionals for greater clinical recognition of the disease are necessary in southern RS, Brazil.
